# Evolutionary implications of heterochromatin and rDNA in chromosome number and genome size changes during dysploidy: A case study in *Reichardia* genus

**DOI:** 10.1371/journal.pone.0182318

**Published:** 2017-08-09

**Authors:** Sonja Siljak-Yakovlev, Bernard Godelle, Vlatka Zoldos, Joan Vallès, Teresa Garnatje, Oriane Hidalgo

**Affiliations:** 1 Ecologie Systématique Evolution, CNRS, AgroParisTech, Univ. Paris-Sud, Université Paris-Saclay, Orsay, France; 2 Institut des Sciences de l’Evolution (CNRS-UMR 5554), Université Montpellier II, Place Eugène Bataillon, Montpellier, France; 3 Department of Biology, Division of Molecular Biology, University of Zagreb, Faculty of Science, Zagreb, Croatia; 4 Laboratori de Botànica (UB) - Unitat associada al CSIC, Facultat de Farmàcia i Ciències de l’Alimentació, Universitat de Barcelona, Barcelona, Catalonia, Spain; 5 Institut Botànic de Barcelona (IBB-CSIC-ICUB), Barcelona, Catalonia, Spain; 6 Comparative Plant & Fungal Biology, Royal Botanic Gardens, Kew, Richmond, Surrey, United Kingdom; Università di Pisa, ITALY

## Abstract

In this study we showed that constitutive heterochromatin, GC-rich DNA and rDNA are implicated in chromosomal rearrangements during the basic chromosome number changing (dysploidy) in *Reichardia* genus. This small Mediterranean genus comprises 8–10 species and presents three basic chromosome numbers (*x* = 9, 8 and 7). To assess genome evolution and differentiation processes, studies were conducted in a dysploid series of six species: *R*. *dichotoma*, *R*. *macrophylla* and *R*. *albanica* (2*n* = 18), *R*. *tingitana* and *R*. *gaditana* (2*n* = 16), and *R*. *picroides* (2*n* = 14). The molecular phylogeny reconstruction comprised three additional species (*R*. *crystallina* and *R*. *ligulata*, 2*n* = 16 and *R*. *intermedia*, 2*n* = 14). Our results indicate that the way of dysploidy is descending. During this process, a positive correlation was observed between chromosome number and genome size, rDNA loci number and pollen size, although only the correlation between chromosome number and genome size is still recovered significant once considering the phylogenetic effect. Fluorescent *in situ* hybridisation also evidenced changes in number, position and organisation of two rDNA families (35S and 5S), including the reduction of loci number and, consequently, reduction in the number of secondary constrictions and nuclear organising regions from three to one per diploid genome. The potential mechanisms of chromosomal and genome evolution, strongly implicating heterochromatin, are proposed and discussed, with particular consideration for *Reichardia* genus.

## Introduction

The Mediterranean genus *Reichardia* Roth (Asteraceae), made up of annual, biennial or perennial herbs, is a good model for the investigation of genome organisation and evolution since it includes a small number of closely related species (from 8 to 10, depending on the authors) and three basic chromosome numbers: *x* = 9, 8, and 7. Two tertiary relict species, one from Dinaric Alps and another from Middle East (*R*. *macrophylla* Vis. & Pančić and *R*. *dichotoma* (DC.) Freyn (*R*. *glauca* V.A.Matthews), respectively), have *x* = 9, in common with the recently described Albanian endemic *R*. *albanica* F.Conti & D.Lakušić, which is closely related to the latter taxa [1]. During the quaternary glaciations, certain regions of the Dinaric Alps became refugia for Tertiary flora; some habitats on dolomite substrate are exceptionally rich in endemic or relict species, among which *R*. *macrophylla* is included [2, 3]. The second relict species is *R*. *dichotoma*, whose geographical distribution is limited to the Eastern Mediterranean (Anatolia, Armenia, Georgia, North-East Iran, Syria and North Lebanon) [1]. *Reichardia picroides* (L.) Roth and *R*. *intermedia* (Sch.Bip.) Cout., with circum-Mediterranean distribution [4], have the lowest basic chromosome number in the genus, *x* = 7. In this dysploid series other species such as *R*. *tingitana* (L.) Roth, with a repartition from the Azores to NW India [5] (which coincides with paleogeographical limits of the Mediterranean basin [6]), the Iberian neoendemic species *R*. *gaditana* (Willk.) Cout. [7], and three endemic species from Canary Islands (*R*. *crystallina* (Sch.Bip.) Bramwell, *R*. *famarae* Bramwell & G.Kunkel ex M.J.Gallego & Talavera and *R*. *ligulata* (Vent.) G.Kunkel & Sunding), have an intermediate basic number of *x* = 8 [5, 8, 9, 10].

According to *Flora Europaea*, the endemic species from Balkan Peninsula *R*. *macrophylla* has been considered to be *R*. *picroides* [11]. However, these two taxa have different basic chromosome numbers of *x* = 9 and *x* = 7, respectively [12], and different geographical ranges. *Reichardia picroides* has a large circum-Mediterranean repartition, while *R*. *macrophylla* grows in regions considered refugia of Tertiary flora, such as canyons and narrow dry valleys on limestone or dolomite substrata, frequently in *Pinus nigra* J.F.Arnold communities [2, 3].

Genome size, usually assessed as the 2C value (the amount of DNA in a somatic unreplicated nucleus) [13, 14], is one of the most relevant biological characters, with relationships with many other plant life characters, from morphological to ecological through cytogenetic, phylogenetic and even taxonomical ones [15] (and references therein). Relationships between nuclear DNA amount and chromosomal characters are numerous and clear in the Asteraceae family [16] (and references therein). Of the consequences of genome size in morphological traits, pollen size has been largely understudied, except in the case of the species with different ploidy levels [17].

Among plant species, there is great variability in chromosome number, with variation of basic chromosome number ("*x*") across a wide range [18] (and references therein). Amongst the rare intra-specific variations, the most frequent are the modifications of ploidy level (very frequent in plants) or Robertsonian mutations. Increases in ploidy level seem to be produced by naturally occurring mutations causing extensive genome rearrangements, resulting in modifications of life cycle, such as flowering time [19], which might lead in some cases to the rise of new species. Although polyploidy is a well-known evolutionary mechanism in plants, in some cases the main evolutionary trend is not a genome multiplication, but a progressive reduction of the basic number, known as dysploidy, namely decreasing, descending or downward dysploidy (from *x* to *x*-1, *x*-2, *x*-3 etc.) [10, 20, 21].

We previously studied karyotype and constitutive heterochromatin patterns in five of the above-mentioned species of this genus by Giemsa C-banding [10, 22, 23]. There is almost no heterochromatin in *R*. *dichotoma* and only a tiny heterochromatic band in *R*. *macrophylla*. In contrast, the increase in presence of heterochromatin was observed in two species with *x* = 8, *R*. *gaditana* and *R*. *tingitana*, which possess even entire heterochromatic short arms of some chromosome pairs. In *R*. *picroides*, whose basic chromosome number is reduced to *x* = 7, the decrease in heterochromatin and its restriction to centromeres and secondary constrictions (SC) were detected. A possible way to test the role of heterochromatin in governing chromosomal rearrangements during reduction of chromosome number and its impact on genome size changes requires considering closely related species with differentiated karyotypes such as is the case in the genus *Reichardia*.

Heterochromatin is frequently associated with chromosomal rearrangements [24, 25, 26, 27]. However, it remains to be examined whether it can act upon these rearrangements by making them more likely or less deleterious, considering its well-known properties and its distribution along chromosomes in the *Reichardia* species.

Heterochromatin is an important constituent of the genome and could be highly informative for untangling the evolutionary histories of closely related species. Constitutive heterochromatin is made up of tandemly repeated sequences which can be AT or GC rich [28, 29, 30] and can be revealed by several stain procedures, such as Giemsa C- [31] and fluorochrome banding [32, 33]. Several authors, especially Schweizer et al. [34], suggest that heterochromatin modification is a rapid process compared to other biological processes ("recent icing on the cake"). At the karyotypic level, it can modify meiotic recombination [26, 35, 36, 37, 38, 39], form interchromosomal connections [10], and affect variation in genome size. In addition, it is frequently associated with translocations, inversions or chromosomal breaks and involved in chromosome segregation [26, 40].

Molecular cytogenetic techniques provide the opportunity to study the fine mechanisms that have acted during the evolution of the chromosome, e.g. dysploidy. In eukaryotes, the rRNA genes can serve as excellent markers in phylogenetic studies. These genes are organised into two distinct families (i.e., 35S and 5S rDNA) that occur as tandem arrays at one or more specific chromosomal regions. Due to their high copy number, detection of the rRNA genes is highly reproducible and provides valuable information concerning chromosomal evolution. Copy number and chromosomal distribution of rDNAs can change rapidly and rDNA transposition or dispersion in plant genomes is observed frequently [41, 42, 43, 44, 45, 46, 47]. These rearrangements generally correlate with species differentiation and speciation. The numbers and locations of rDNA arrays may vary even between infra-specific taxa and can therefore provide chromosomal landmarks for species differentiation [48].

Based on our previous karyological studies [10, 12, 23] we postulated the hypothesis about descending dysploidy in the genus *Reichardia*. Thus, the main objective of the present work was to understand the mechanism of karyotype evolution by dysploidy in a small cluster of closely related species by checking possible heterochromatin involvement. For this purpose, karyotypes of *Reichardia* species were characterised using molecular cytogenetic techniques: (1) flow cytometry for DNA quantity and GC% assessment; (2) fluorochrome banding for distribution of GC-rich DNA and neutral heterochromatin; (3) FISH for establishing a physical map of 35S (18S-5.8S-26S) and 5S rRNA genes. In addition, pollen grain size variation relative to basic chromosome number and genome size was measured. The data obtained were analysed in the frame of new molecular phylogenetic evidence.

## Material and methods

### Origin of material

The origins of the studied populations are shown in Table 1. Six out of the nine species studied are endemics: *R*. *gaditana* (Iberian Peninsula), *R*. *macrophylla* (Dinaric Alps), *R*. *albanica* (from Albania) and *R*. *dichotoma* (from the Middle East), plus two endemic species from the Canary Islands, *R*. *crystallina* and *R*. *ligulata*. The remaining species are more widespread: *R*. *tingitana* from the Azores to NW India, and *R*. *picroides* and *R*. *intermedia* across the whole Mediterranean basin.

**Table 1:** Origin of studied species and populations. Vouchers are deposited in the following herbaria. BCN: Centre de Documentació de Biodiversitat Vegetal, Universitat de Barcelona. BC: Institut Botànic de Barcelona. BEOU: University of Belgrade. SY: Sonja Siljak-Yakovlev (personal collection), Orsay.

Species	Locality	Collectors and herbarium where voucher is deposited
*R*. *dichotoma* (DC.) Freyn (*R*. *glauca* A.Matthews)	1. Mountain pass Tigranashen and Sovetashen, Armenia	G. Fajvush, E. Gabrielian, N. Garcia-Jacas, M. Hovanyssian, A. Susanna, J. Vallès (BCN)
	2. Marand, Iran	N. Garcia-Jacas, A. Susanna, V. Mozaffarian, J. Vallès (BCN)
	3. Mt Ehden, Lebanon	S. Siljak-Yakovlev, M. Bou Dagher-Kharrat, (SY)
*R*. *macrophylla* Vis. & Pančić	4. Near Konjic, Bosnia & Herzegovina	S. Siljak-Yakovlev (SY)
	5. Diva Grabovica, Bosnia & Herzegovina	
	6. Mt Orjen, Montenegro	
	7. Lastva, Bosnia & Herzegovina	
	8. Sutjeska canyon, Bosnia & Herzegovina	
*R*. *albanica* F. Conti & D. Lakušić	9. Mali i Cikes, Llogara, Albania	D. Lakušić, N. Kuzmanović, M. Lazarevic, A. Alegro, F. Conti (BEOU)
*R*. *tingitana* (L.) Roth	10. Canary Islands, Spain	From Puerto de la Cruz botanic garden (SY)
	11. Oriola, Spain	J. Vallès (BCN)
*R*. *gaditana* (Willk.) Cout.	12. Portugal	M. Queirós (from Coimbra botanical garden) (SY)
*R*. *crystallina* (Sch.Bip.) Bramwell	13. Porís de Abona,Tenerife, Canary Islands, Spain	A. Santos-Guerra, J. Vallès (BCN)
*R*. *ligulata* (Vent.) G.Kunkel & Sunding	14. Punta de Teno,Tenerife, Canary Islands, Spain	A. Santos-Guerra, J. Vallès (BCN)
	15. Roque de las Bodegas, Tenerife, Canary Islands, Spain	A. Santos-Guerra, J. Vallès (BCN)
	16. Andén Verde, Gran Canaria, Canary Islands, Spain	A. Santos-Guerra, J. Vallès (BCN)
*R*. *intermedia* (Sch.Bip.) Cout.	17. Oran, Algeria	K. Abdeddaim (SY)
	18. Spain	T. Garnatje (from Barcelona botanical garden) (BC)
*R*. *picroides* (L.) Roth	19. Gornji Okrug, Dalmatia, Croatia	S. Siljak-Yakovlev (SY)
	20. Dubrovnik, Dalmatia, Croatia	
	21. Lavandou, Côte d’Azur, France	

From cytogenetic and palynological points of view we have studied five well representative species, comprising all three basic chromosome numbers, among the nine species of genus. Out of the four not studied from this viewpoint, three are endemic to the Canary Islands and are close to *R*. *tingitana* (a nearly circum-Mediterranean species with 2*n* = 16), and one (*R*. *intermedia*) is very close to *R*. *picroides* (2*n* = 14). For molecular phylogenetic study and genome size estimation all species of the genus were considered except *Reichardia famarae* Bramwell & G.Kunkel ex Gallego & Talavera, endemic from Canary Islands, for which we missed the material.

### Estimation of nuclear DNA content and base composition by flow cytometry

Total DNA amounts were assessed by flow cytometry according to Marie and Brown [49]. *Petunia hybrida* Vilm. ‘PxPc6’ (2C = 2.85 pg, 41.0% GC) and *Lycopersicon esculentum* Mill. ‘Roma’ (2C = 1.99 pg, 40.0% GC) were used as internal standards. Leaves of both the studied species and the internal standard were chopped up using a razor blade in a plastic Petri dish with 600 μl of Galbraith nucleus-isolation buffer [50] containing 0.1% (w/v) Triton X-100, 10 mM sodium metabisulphite and 1% polyvinylpyrrolidone 10,000. The suspension was passed through a 48 μm mesh nylon filter. The nuclei were stained with 50 μg/ml propidium iodide, after 15 min RNase treatment (2.5 U/ml). Base composition was assessed using AT-specific fluorochrome bisbenzimide Hoechst 33342 (5 μg/ml; Aldrich) and GC-specific fluorochrome mithramycin (50 μg/ml). DNA content of 5,000–10,000 stained nuclei was determined for each sample using an Elite ESP flow cytometer (Beckman-Coulter, Roissy, France) with a water-cooled argon laser. Total 2C DNA value was calculated using the linear relationship between the fluorescent signals from the stained nuclei of the *Reichardia* specimen and the internal standard. Base composition (GC percentage) was calculated using the nonlinear model established by Godelle et al. [51]. Each studied population comprised at least five individuals, measured separately and with two replicates.

### Chromosome preparation

Root tips obtained from germinated seedlings or from living plants (growing in experimental garden, Orsay, France), were pre-treated with 2 mM 8-hydroxyquinoline during 2 h (*R*. *picroides* and *R*. *gaditana*), 2 h 15 min (*R*. *tingitana*) or 3 h (*R*. *macrophylla*, *R*. *dichotoma* and *R*. *albanica*) at approximately 16°C, and then fixed in 3:1 absolute ethanol:glacial acetic acid at 4°C for at least one day. Chromosome plates for fluorochrome banding and FISH experiments were prepared using the air-drying technique of Geber and Schweizer [52], with slight modifications. Root tips were washed in citrate buffer (pH 4.6) for 10 min and then transferred into the enzyme mixture [4% cellulase “Onozuka” R-10 (Yakult Honsha Co. Tokyo, Japan), 1% pectolyase Y-23 (Seishin Co. Tokyo, Japan), 4% hemicellulase (Sigma)] at 37°C for 15 min. The resulting protoplast suspension was washed three times in citrate buffer and fixed in 3:1 absolute ethanol:glacial acetic acid. Centrifugation was performed at 4000 rpm (1500 g) for 5 min. The final pellet was resuspended in 50 μl of fixative and protoplasts were transferred onto a clean slide, air-dried, and kept at room temperature until use.

### Fluorochrome banding and rDNA mapping by fluorescent *in situ* hybridisation (FISH)

GC-rich DNA region staining with chromomycin A_3_ (CMA_3_, Sigma Aldrich Co., Steinheim, Germany) was performed according to Schweizer [32] with minor modifications as described by Siljak-Yakovlev et al. [33].

A double FISH experiment was carried out following the method of Heslop-Harrison et al. [53]. Slides were counterstained and mounted in Vectashield medium containing DAPI (4′, 6-diamidino-2-phenylindole, Vector Laboratories) which also revealed neutral or nonspecific heterochromatin (rich neither in AT nor in GC bases) as DAPI^+^ bands. This type of heterochromatin mainly corresponded to constitutive heterochromatin stained by Giemsa C-banding.

For rDNA analyses and CMA fluorochrome banding, a minimum of 10 well-spread metaphases were analysed for each species.

### Microscopy and chromosome analysis

Chromosome observations were performed using an epifluorescence Zeiss Axiophot microscope with different combinations of excitation and emission filter sets (01, 07, 15 and triple filter set 25). The signals were analysed using the highly sensitive CCD camera (RETIGA 2000R; Princeton Instruments, Evry, France) and an image analyser (Metavue, Evry, France).

The construction of idiograms and the Giemsa C-banding for detection of constitutive heterochromatin in five representatives have been published in two our previous works [10, 23].

### Pollen grain measurement

Pollen grains were acetolysed according to Erdtman [54]. The measurements of pollen grains’ polar axis (P) and equatorial (E) diameter were performed on 100 acetolysed grains mounted for at least three weeks in glycerine jelly. All measurements were made on well-formed pollen grains under a 40× objective lens on Zeiss Axiophot microscope.

### DNA extraction, amplification and sequencing

Total genomic DNA was extracted following the CTAB method of Doyle and Doyle [55] as modified by Soltis et al. [56], from silica gel-dried leaves collected in the field or fresh leaves of plants cultivated in the Botanical Institute of Barcelona. In some cases, herbarium material was used. Double-stranded DNA was amplified from ITS regions with the 1406F [57] and ITS4 [58] primers. In some cases, we used the ITS1 [58] as forward primer. PCR products were purified with the QIAquick PCR purification kit (Qiagen, Valencia, California, U.S.A.). Both strands were sequenced with 1406F or ITS1 as forward primers and ITS4 as the reverse primer. Direct sequencing of the amplified DNA segments was performed using Big Dye Terminator Cycle sequencing v2.0 (PE Biosystems, Foster City, California, U.S.A.). Nucleotide sequencing was carried out at the Centres Científics i Tecnològics, University of Barcelona on an ABI PRISM 3700 DNA analyser (PE Biosystems, Foster City, California, U.S.A.).

DNA sequences were edited with Chromas 1.56 (Technelysium PTy, Tewantin, Queensland, Australia) and aligned visually. The sequences were deposited in GenBank (see the Appendix for the accession numbers). The sequence alignment is available from the corresponding author.

### Phylogenetic analysis

To determine model under the Akaike Information Criterion (AIC) [59] the data set was analysed using MrModeltest 2.2 [60]. This model was used to perform a Bayesian analysis using MrBayes 3.2.1 [61]. Four Markov chains were run simultaneously for two million generations, and these were sampled every 100 generations. Data from the first 1000 generations were discarded as the burn-in period, after confirming that likelihood values had stabilised prior to the 1000^th^ generation. Posterior probabilities were estimated through the construction of a 50% majority rule consensus. The outgroup (*Sonchus kirkii* Hamlin) has been chosen on the basis of the work of Kim et al. [62].

### Ancestral character states reconstructions

The *Phytools* package of R [63] was used to perform ancestral state reconstructions, using the consensus tree resulting from Bayesian analysis reduced to the set of ingroup taxa. The ancestral 2C-values were reconstructed under maximum likelihood with the *fastAnc* and *contMap* commands, and ancestral chromosome numbers were inferred with the re-rooting method. Alternatively, ancestral GS were also reconstructed using maximum parsimony for continuous traits in Mesquite v.3.04 software [64].

### Correlation analyses

Phylogenetic generalised least squares analyses (PGLS) were conducted under the Brownian motion model and Pagel model of evolution using the ape and *nlme* packages of R [65, 66, 67] on the log-transformed dataset of Bayesian tree, ultrametricised and pruned to the five species with available data for all traits.

## Results

### Genome size

The genome size of the studied populations ranged from 2.86 (*R*. *picroides*) to 5.54 pg (*R*. *dichotoma*) for holoploid (2C) DNA (or from 1399 to 2709 Mbp for monoploid genome size (1Cx). Coefficients of variation were under 5, accounting for a reliable quality of the measurements (Table 2). These data were in agreement with the basic chromosome number: species with *x* = 9 showed the highest DNA content (mean value of 5.21 pg/2C) and those with *x* = 8 and *x* = 7 showed lower DNA content (mean values of 3.52 and 2.92 pg/2C, respectively). The percent of G-C bases (GC %) ranges from 39.4 in *R*. *picroides* to 41.2 in *R*. *gaditana* (Table 2).

**Table 2:** DNA content and GC percentage of *Reichardia* species from different populations.

Species (2*n*)	Population number (see Table 1) or name	2C DNA in pg^a^ (SD; CV)	1C*x* in Mbp [68]	GC%
*R*. *dichotoma* (18)	1	5.20 (0.04; 1.23)	2543	40.7
	2	5.08 (0.03; 0.79)	2484	40.4
	3	5.54 (0.07; 0.83)	2709	
Mean for species		***5*.*27***		***40*.*6***
*R*. *macrophylla* (18)	4	5.25 (0.04; 3.66) [69]	2567	
	5	5.13 (0.10; 0.77)	2509	
	6	5.20 (0.09; 0.75)	2543	
	7	5.10 (0.10; 0.66) [70]	2494	40.9
	8	5.05 (0.07; 0.79)	2469	
Mean for species		***5*.*15***		
*R*. *albanica* (18)	9	5.22 (0.02; 0.43) [1]	2553	
*R*. *tingitana* (16)	10	3.50 (0.07; 0.83) [70]	1712	40.5
	11	3.23 (0.02; 0.58)	1579	
Mean for species		***3*.*37***		
*R*. *gaditana* (16)	12	3.40 (0.08; 0.67) [70]	1663	41.2
*R*. *crystallina* (16)	13	3.43 (0.08; 2.35) [71]	1677	-
*R*. *ligulata* (16)	14	3.84 (0.32; 2.42) [71]	1878	
	15	3.62 (0.14; 2.29) [71]	1770	
	16	3.95 (0.50; 2.73) [71]	1932	
	Barranco de Roque	3.78 (0.01; 2.21) [72]	1852	
	Bermejo [72]			
Mean for species		***3*.*80***		
*R*. *intermedia* (14)	17	2.90 (0.02; 1.11) [70]	1418	
	18	2.93 (0.04; 1.24)	1433	
Mean for species		***2*.*92***		
*R*. *picroides* (14)	19	2.90 (0.05; 0.45)	1418	39.4
	20	3.00 (0.03; 0.78) [70]	1467	39.5
	21	2.86 (0.02; 0.38)	1399	39.9
Mean for species		***2*.*92***		***39*.*6***

^a^Mean value for population; SD = standard deviation; CV = coefficient of variation

### Mapping of heterochromatin and rRNA genes

#### Neutral (unspecific) heterochromatin pattern (DAPI^+^ bands)

**Fig 1:**
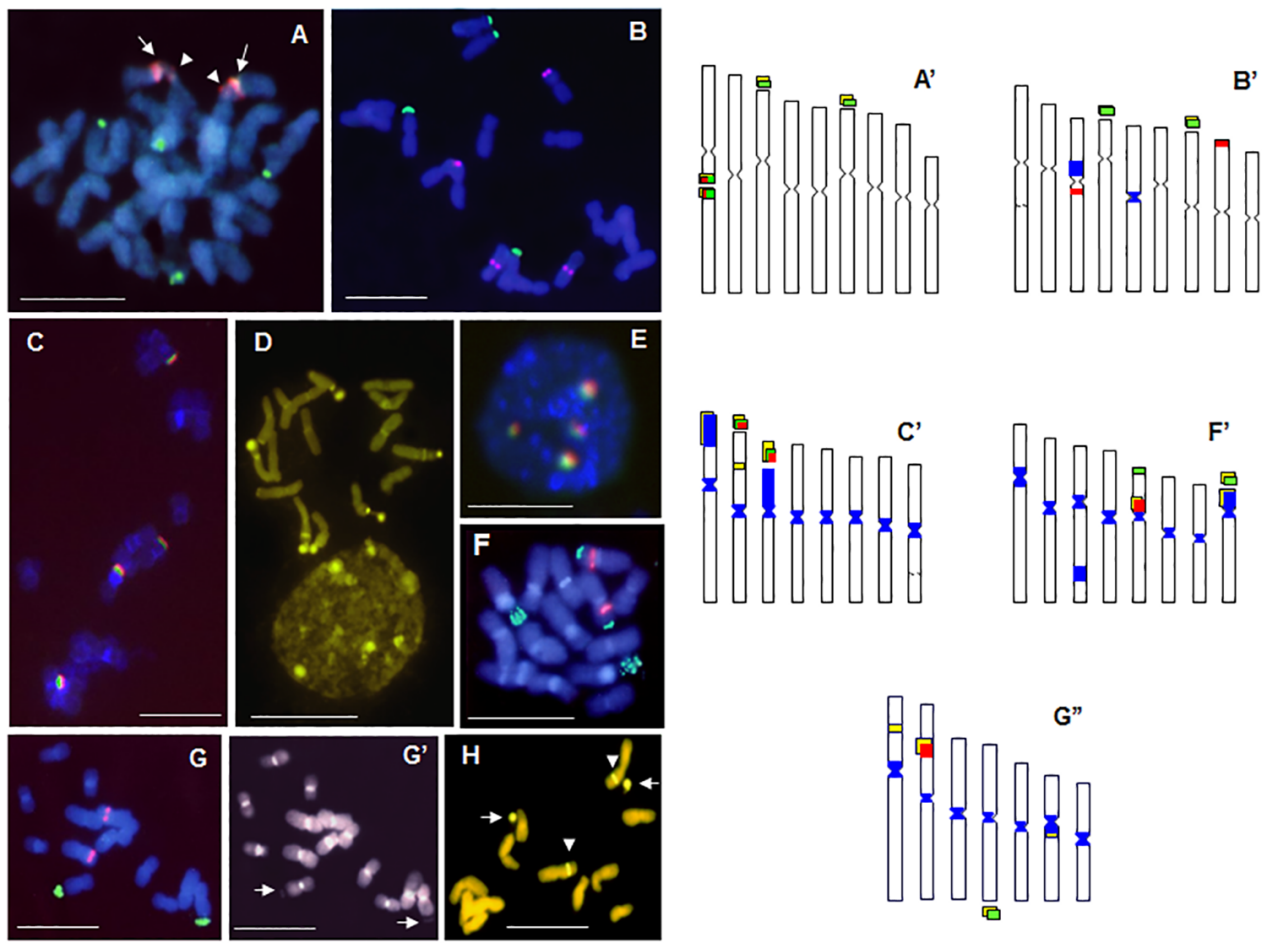
Metaphase chromosome plates and interphase nuclei of *Reichardia* species after double target FISH with 5S (red signals) and 35S (green signals) rDNA loci, CMA (yellow signals) and DAPI staining (blue): *R*. *dichotoma* showing 5S and 35S colocalised loci at both sites of intercalary secondary constriction (arrows and arrowheads) and four terminal 35S signals (A); *R*. *macrophylla* with 4 terminal 35S signals and two intercalary and two terminal 5S signals (B); *R*. *tingitana* showing four colocalised 5S and 35S signals (C), numerous CMA^+^ bands in metaphase chromosomes and interphase nucleus (D), nucleus after FISH with four 35S/5S spots (E); *R*. *gaditana* with four terminal 35S (two being very intense) and two intercalary 5S signals (F); *R*. *picroides* showing two terminal sat 35S on long chromosome arms and two intercalary 5S signals on short arms (G), DAPI^+^ centromeric bands and DAPI negative satellites (arrows) in the same metaphase plate as FISH, better visible on black and white photograph (G’) and CMA^+^ satellites (arrows) corresponding to 35S and intercalary bands (arrowheads) that corresponds to 5S signals (H). Scale bar 10 *μ*m. Idiograms of *R*. *dichotoma* (A’), *R*. *macrophylla* (B’), *R*. *tingitana* (C’), *R*. *gaditana* (F’), *R*. *picroides* (G”) showing distribution of CMA^+^ (yellow) and DAPI^+^ (blue) bands, 35S (green) and 5S (red) rDNA signals.

The band positions of neutral (or unspecific) heterochromatin, revealed by DAPI after FISH, are shown in Table 3 and Fig 1. In *R*. *dichotoma* this type of heterochromatin was not observed (Fig 1A’). In *R*. *macrophylla*, the presence of neutral heterochromatin was restricted to two chromosome pairs; a large paracentromeric band on short arm of pair 3 and a centromeric band on pair 5 (Fig 1B’). In *R*. *tingitana* all the centromeres are heterochromatic, and also a part (pair 1) or the totality (pair 3) of the short arms (Fig 1C’). In *R*. *gaditana*, not only the centromeres, but also one intercalary region (pair 3) and the whole short arm of pair 8 are stained (Fig 1F and 1F’). The unspecific heterochromatin only remains in centromeres of *R*. *picroides* (Fig 1G’ and 1G”).

**Table 3:** Distribution of DAPI bands after FISH experiment.

Species (2*n*)	Centromeric region	Pericentromeric region	Paracentromeric region	Intercalary band	Terminal band
*R*. *dichotoma* (18)	0	0	0	0	0
*R*. *macrophylla* (18)	pairs 3, 5	0	pair 3	0	0
*R*. *tingitana* (16)	all pairs	pair 3^a^	0	0	pair 1
*R*. *gaditana* (16)	all pairs	pair 1	0	pairs 3, 8^b^	0
*R*. *picroides* (14)	all pairs	0	pairs 1, 6	0	0
*R*. *intermedia* (14)	all pairs	0	pairs 1, 6	0	0

pair = chromosome pair,

^a^entire DAPI positive chromosome arm comprising secondary constriction,

^b^entire DAPI positive chromosome arm except satellite

#### G-C rich heterochromatin distribution (CMA^+^ bands)

The number and the distribution of CMA^+^ bands are presented in Table 4. Chromomycin positive bands were visible in all satellites and secondary constrictions (Fig 1). Additional terminal or intercalary bands were observed in *R*. *tingitana* (Fig 1D and 1C’), *R*. *gaditana* (Fig 1F) and *R*. *picroides* (Figs 1H and 1G”).

**Table 4:** Number and distribution of CMA^+^ bands in diploid chromosome set.

Species (2*n*)	Total CMA^+^ bands number	Intercalary bands in SC	Terminal bands	Satellite SC	Intercalary bands	PC bands
*R*. *dichotoma* (18)	6	pair 1	0	pairs 3, 6	0	0
*R*. *macrophylla* (18)	4	0	0	pairs 4, 7	0	0
*R*. *tingitana* (16)	8	0	pairs 1, 3	pair 2	pair 2	0
*R*. *gaditana* (16)	6	0	pair 8*	pair 8	0	pair 5
*R*. *picroides* (14)	8	0	0	pair 4	pairs 1, 2	pair 6
*R*. *intermedia* (14)	8	0	0	pair 4	pairs 1, 2	pair 6

pair = chromosome pair,

*entire short chromosome arm,

PC = paracentromeric bands

#### Physical mapping of 35 and 5S rRNA genes

The number and position of 35S and 5S rDNA loci are shown in Table 5. In *R*. *dichotoma* there were three 35S rDNA loci (two in satellite and one in intercalary SC) and one 5S rDNA locus (Fig 1A and 1A’). The only 5S rRNA site was colocalised with the 35S in intercalary SC on the long arm of chromosome pair 1. Both 35S and 5S signals were present on both sides of this SC (Fig 1A and 1A’). In the second species with *x* = 9, *R*. *macrophylla*, two 35S rDNA loci, both in satellite SCs, and also two 5S loci, one on intercalary position near the centromere on the long arm of the pair 3, and another terminal on the short arm of pair 8, were observed (Fig 1B and 1B’). *Reichardia tingitana* displayed four colocalised 5S and 35S signals in metaphase chromosomes (Fig 1C and 1C’) and four 35S/5S signals in nucleus (Fig 1E).

**Table 5:** Number and position of 35S and 5S rDNA loci.

Species (2*n*)	35S rDNA loci		5S rDNA loci	
	number	position	number	position
*R*. *dichotoma* (18)	3	pair 1 –intercalary on both sides of SC;pairs 3 and 6 –sat SCs	1	pair 1 –intercalary on both sides of SC
*R*. *macrophylla* (18)	2	pairs 4 and 7 –sat SCs	2	pair 3 –intercalary; pair 8 –terminal
*R*. *tingitana* (16)	2	pairs 2 and 3 –sat SCs	2	pairs 2 and 3 –sat SCs
*R*. *gaditana* (16)	2	pair 5 –terminal; pair 8 –sat SC	1	pair 5 –intercalary
*R*. *picroides* (14)	1	pair 4 –sat SC long arm	1	pair 2 –intercalary
*R*. *intermedia* (14)	1	pair 4 –sat SC long arm	1	pair 2 –intercalary

*Reichardia gaditana* possessed one terminal and one satellite 35S locus (pair 5 and 8 respectively) and one 5S locus near the centromere on the short arm of pair 5 (Fig 1F and 1F’). The 5S and one of the 35S sites are positioned on the same chromosome arm. Only one 35S (at the satellite SC of long arm of chromosome pair 4) and one 5S (intercalary on the pair 2) loci were found in *R*. *picroides* (Fig 1G and 1G”). Size dimorphism of the satellites and their detachment from the chromosomes (Fig 1H, arrows), chromomycin bands that correspond to 5S signals (Fig 1H, arrow-heads) and DAPI negative satellites corresponding to 35S signals (Fig 1G’) were also observed.

**Fig 2:**
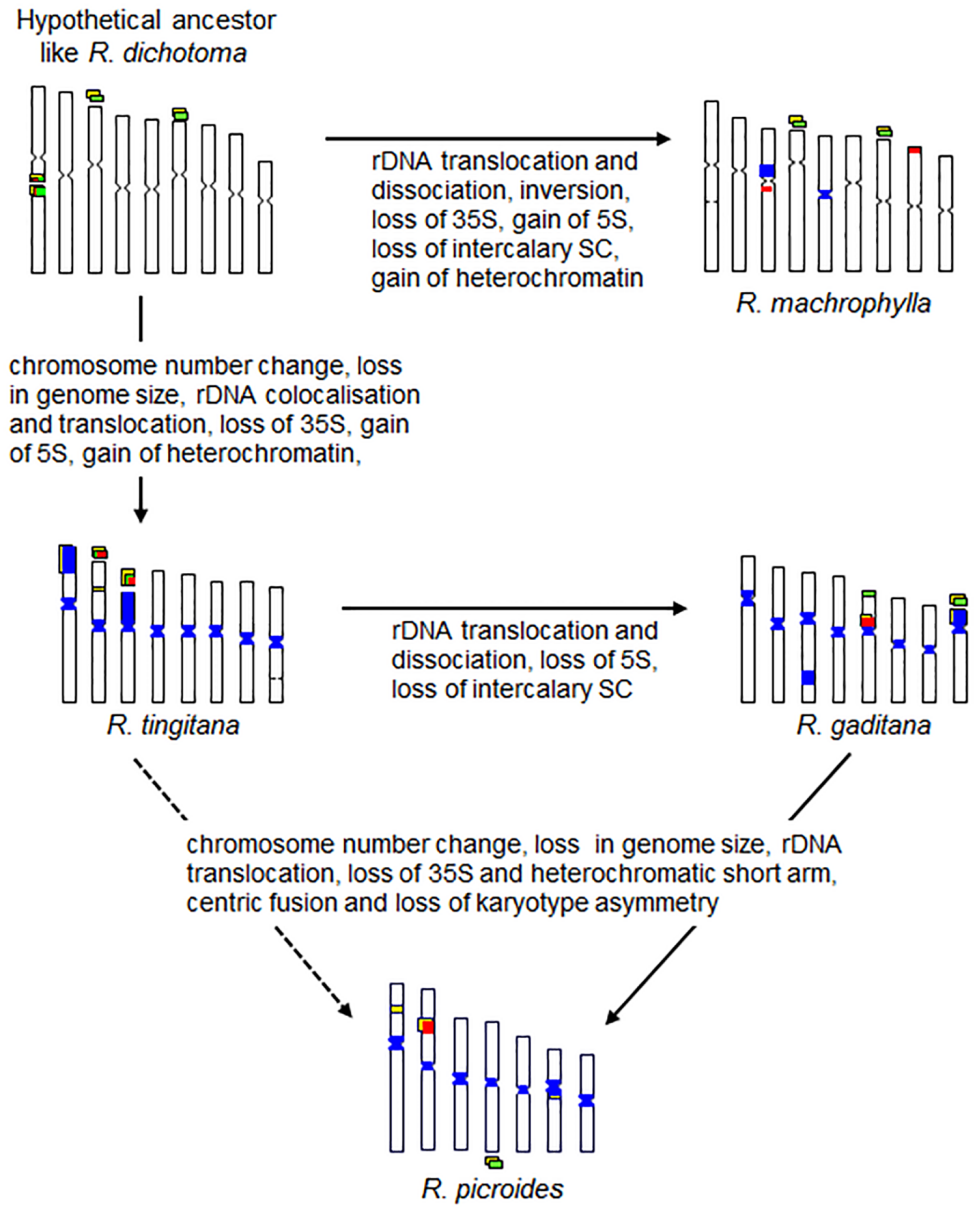
Hypothetical schema of overarching karyotype evolution in the genus *Reichardia* involving heterochromatin, rDNA and genome size changes during descending dysploidy.

According to obtained results one hypothetical schema of karyotype evolution in the genus *Reichardia* is presented in Fig 2.

### Pollen grain dimensions

The mean values with maximal and minimal measures of polar axis (P) and equatorial diameter E are presented in Table 6. Pollen size decreases with the reduction of the basic chromosome number from E = 40.52 and 38.28 μm for two species with *x* = 9 to 31.72 μm for species with *x* = 7.

**Table 6:** Comparison among data concerning genome size, total length of diploid chromosome set, pollen grain dimensions and Giemsa C-bands.

Species	2*n*	2C DNA (pg)	TKL^1^ in μm [23]	Pollen size (μm) [10]		Number of Giemsa C-bands [23]
				E^2^	P^3^	
*R*. *dichotoma*	18	5.27	67.92 (0.35)^4^	40.52 (0.55) 44–39^5^	35.60 (0.50) 38–34	6
*R*. *macrophylla*	18	5.15	83.32 (0.55)	38.28 (0.53) 40–36	32.12 (0.63)37-30	18
*R*. *albanica*	18	5.22	56.60 (0.27)	-	-	-
*R*. *tingitana*	16	3.37	55.32 (0.40)	33.16 (0.42) 35–32	28.16 (0.43) 30–27	24
*R*. *gaditana*	16	3.40	51.54 (0.35)	34.84 (0.39) 36–33	30.04 (0.37) 31–28	16
*R*. *picroides*	14	2.92	51.70 (0.30)	31.72 (0.45) 34–30	26.72 (0.44) 30–25	16

^**1**^TKL = total karyotype length (2*n*);

^**2**^E = pollen equatorial diameter;

^**3**^P = pollen polar axis;

^**4**^standard deviation;

^**5**^Max—Min of E and P.

### Molecular phylogeny, ancestral character states reconstruction and trait correlation

**Fig 3:**
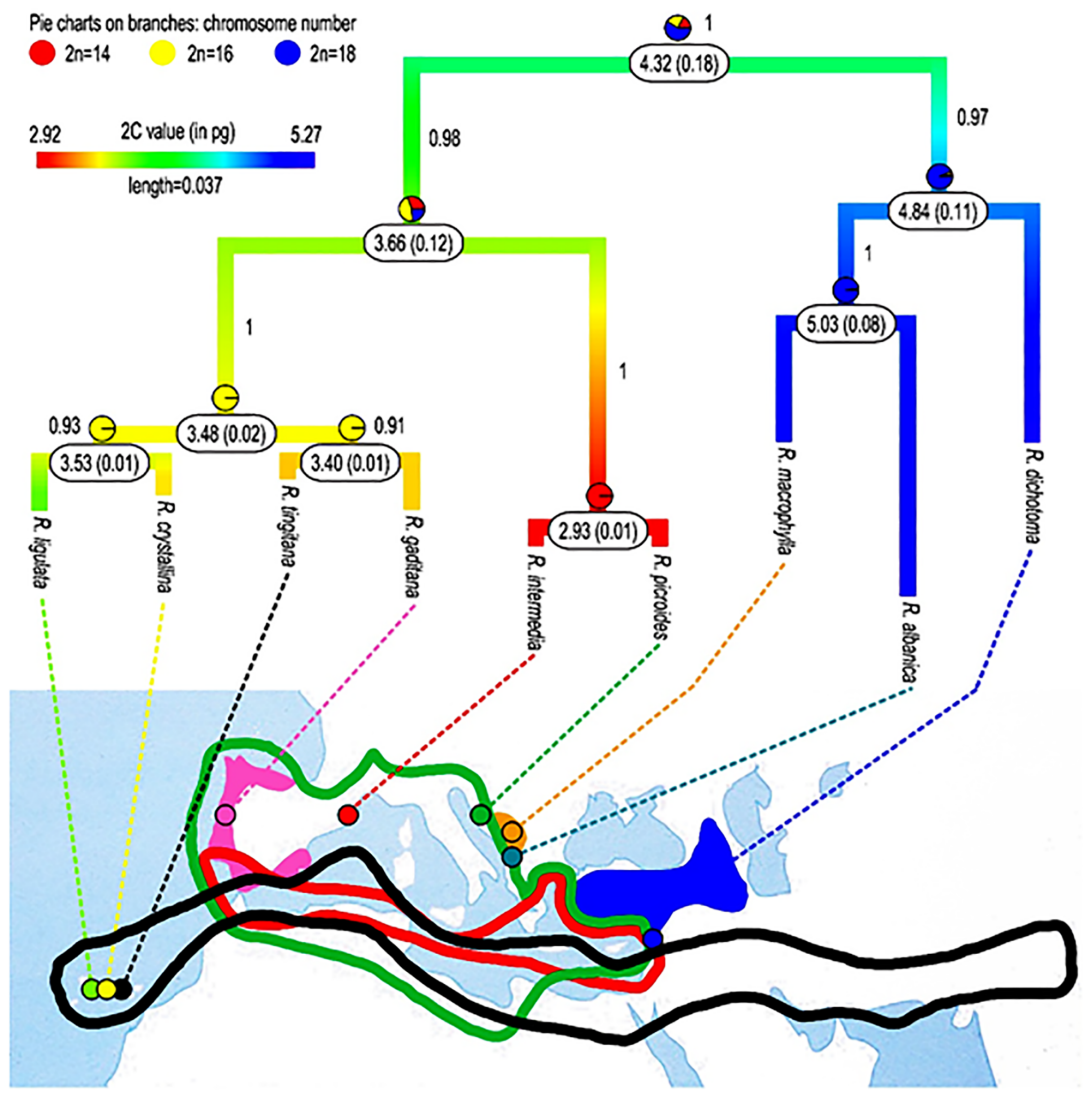
Majority-rule consensus phylogeny of post-burn trees of *Reichardia* obtained through Bayesian analysis of the ITS dataset, plotted on geographic map and showing reconstruction of ancestral genome size and chromosome number. Posterior probabilities are indicated on branches. Values in boxes represent the ancestral genome sizes and their corresponding variances. Dots on the map depict the origin of the sequenced samples. Data on the presence of *Reichardia* species across Mediterranean countries were retrieved from Euro+Med [7] for *R*. *gaditana*, from Blamey and Grey-Wilson [4] for *R*. *intermedia* and for *R*. *macrophylla* and *R*. *albanica* from Conti *et al*. [1].

The phylogenetic tree resulting from the Bayesian analysis of the ITS dataset is presented in Fig 3. It shows the taxa sharing a common chromosome number clustered in well supported clades, with the clade containing the species with *x* = 9 recovered as sister to the two other clades of *x* = 8 and *x* = 7. This topology is compatible with decreasing dysploidy, which was confirmed by the reconstruction of ancestral chromosome numbers (Fig 3). Since ancestral genome size values inferred using Bayesian and parsimony reconstruction methods were similar, we presented here only the results obtained with Bayesian method (Fig 3). From the ancestral state reconstructions, it is noticeable that chromosome number and genome size apparently evolved in parallel. In this sense, a positive and significant relationship between the somatic chromosome number (2*n*) and the holoploid (2C) genome size was supported by the phylogenetic generalised least squares (PGLS) regression analyses (Table 7), regardless of whether the phylogenetic signal was taken into account (*p*_BM_ = 0.0164) or not (*p*_Pagel_ = 0.0145). The positive correlation detected between 2*n* and 35S (*p*_Pagel_ = 0.0464), P (*p*_Pagel_ = 0.0284) and E (*p*_Page_l = 0.0194) loses its significance when considering the phylogenetic signal (Table 7).

**Table 7:** Phylogenetic generalised least squares (PGLS) regression statistics between somatic chromosome number (2n) and other cytogenetic and pollen traits.

	Pagel (λ = 0)		Brownian motion (λ = 1)	
	Slope	*p*	Slope	*p*
CMA	-0.1122	0.4345	-0.2765	0.1402
35S	0.2326	0.0464*	0.1966	0.0683
5S	0.0925	0.5793	0.022	0.7728
TKL	0.4116	0.0864	0.3334	0.2017
2C	0.3649	0.0145*	0.3987	0.0164*
P	0.8672	0.0284*	0.6651	0.1171
E	0.9659	0.0194*	0.7851	0.0946
GC	4.7031	0.2132	2.2225	0.3742
AS	1.2811	0.2472	0.2703	0.6165
R	-0.128	0.5787	-0.3145	0.4849

CMA: chromomycin-positive regions; 35S: 35S rDNA loci; 5S: 5S rDNA loci; TKL: total karyotype length; 2C: holoploid genome size; P: pollen grain polar axis; E: pollen grain equatorial diameter; GC: percentage of GC bases; AS: chromosome asymmetry index; R: chromosome arm ratio. Significance: *P < 0.05.

**Fig 4:**
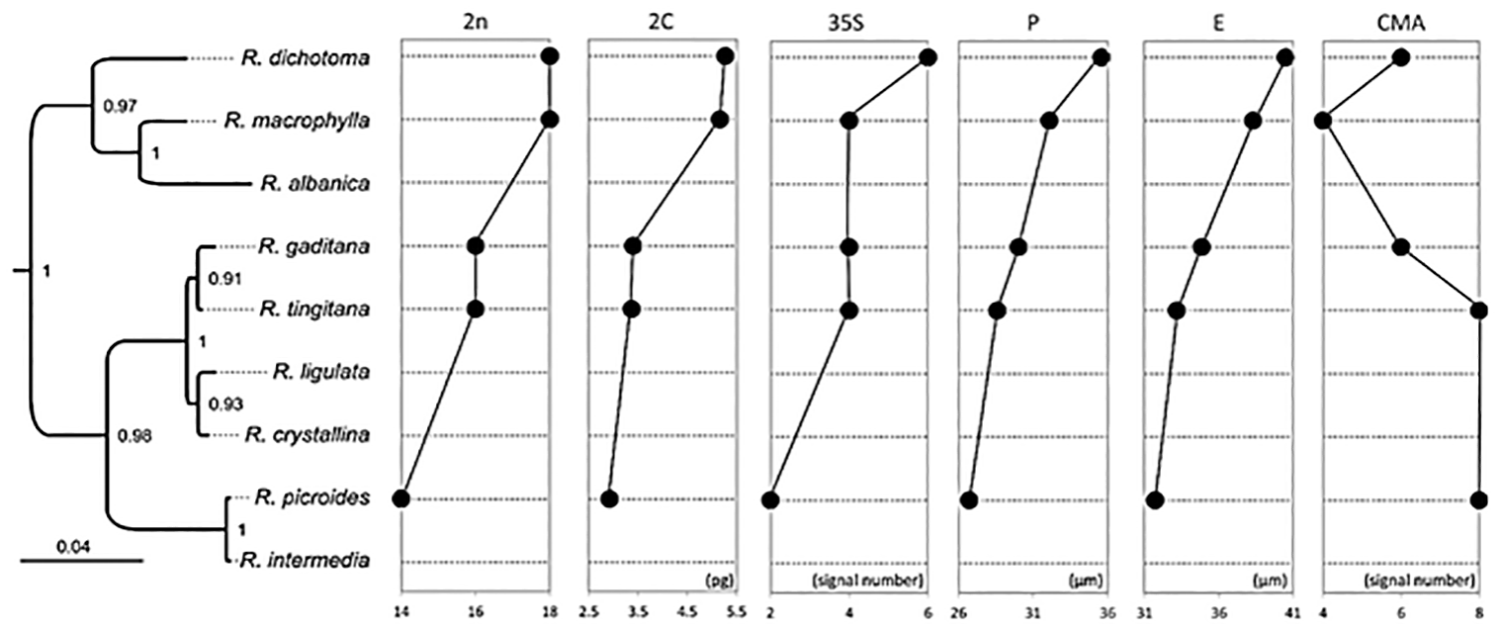
Karyological, cytogenetic and pollen traits plotted on the *Reichardia* phylogeny. Posterior probabilities are indicated on branches.

All our results are summarized on a phylogenetic framework and presented in Fig 4.

## Discussion

### Reduction of basic chromosome number—Descending dysploidy

According to Watanabe’s Index to Plant Chromosome Numbers in Asteraceae (http://www.lib.kobe-u.ac.jp/infolib/meta_pub/G0000003asteraceaeresult-en, accessed August 22^nd^, 2016) [73], the chromosome number (always indicating a diploid level) has already been reported for *R*. *dichotoma* (2*n* = 18), *R*. *macrophylla* (2*n* = 18), *R*. *albanica* (2*n* = 18), *R*. *tingitana* (2*n* = 16), *R*. *gaditana* (2*n* = 16) and *R*. *picroides* and *R*. *intermedia* (2*n* = 14). A few discordant counts, most probably due to plant misidentifications, are also reported.

The basic numbers reported in the five species studied here support the hypothesis of decreasing dysploidy (i.e. progressive reduction of the basic chromosome number) as the most likely mechanism driving the karyotypic evolution in *Reichardia*. Several findings presented here provide further evidence of a reduction of chromosome number, such as:

- The basic number *x* = 9 is generally considered as ancestral in the Asteraceae family [74, 75, 76, 77] and in the Cichorieae tribe [78]. According to Semple and Watanabe [77], *x* = 10 was the ancestral basic number out of the two dominant numbers in the subfamily Cichorioideae, *x* = 10 and *x* = 9; as for the tribe Cichorieae, which *Reichardia* belongs to, only *x* = 9 is indicated as the main basic number by these authors.
- In the genus *Reichardia*, the three species with *x* = 9 are tertiary relicts, their distribution areas being restricted to habitats known as refugia for Tertiary flora [2, 3]. Consequently, the ancestral features are expected in these species.
- *Reichardia picroides*, which presents the lowest basic number (*x* = 7), has a modern distribution area (the whole Mediterranean basin). The low value of its asymmetry index (As) may therefore reflect acquired symmetry or secondary symmetry [23] of the karyotype, resulting from rearrangements during chromosome number reduction.
- The two species with the biggest base chromosome number and genome size (*R*. *dichotoma* and *R*. *macrophylla*) are exclusively perennial, whereas the others are perennial, biennial or annual. In the sister groups of *Reichardia* (*Launaea*, *Sonchus*), annual, biennial and perennial taxa exist as well.
- The inference of ancestral chromosome numbers confirms the descending direction of dysploidy (Fig 3).

### Changes in heterochromatin pattern and ribosomal genes mapping

**Fig 5:**
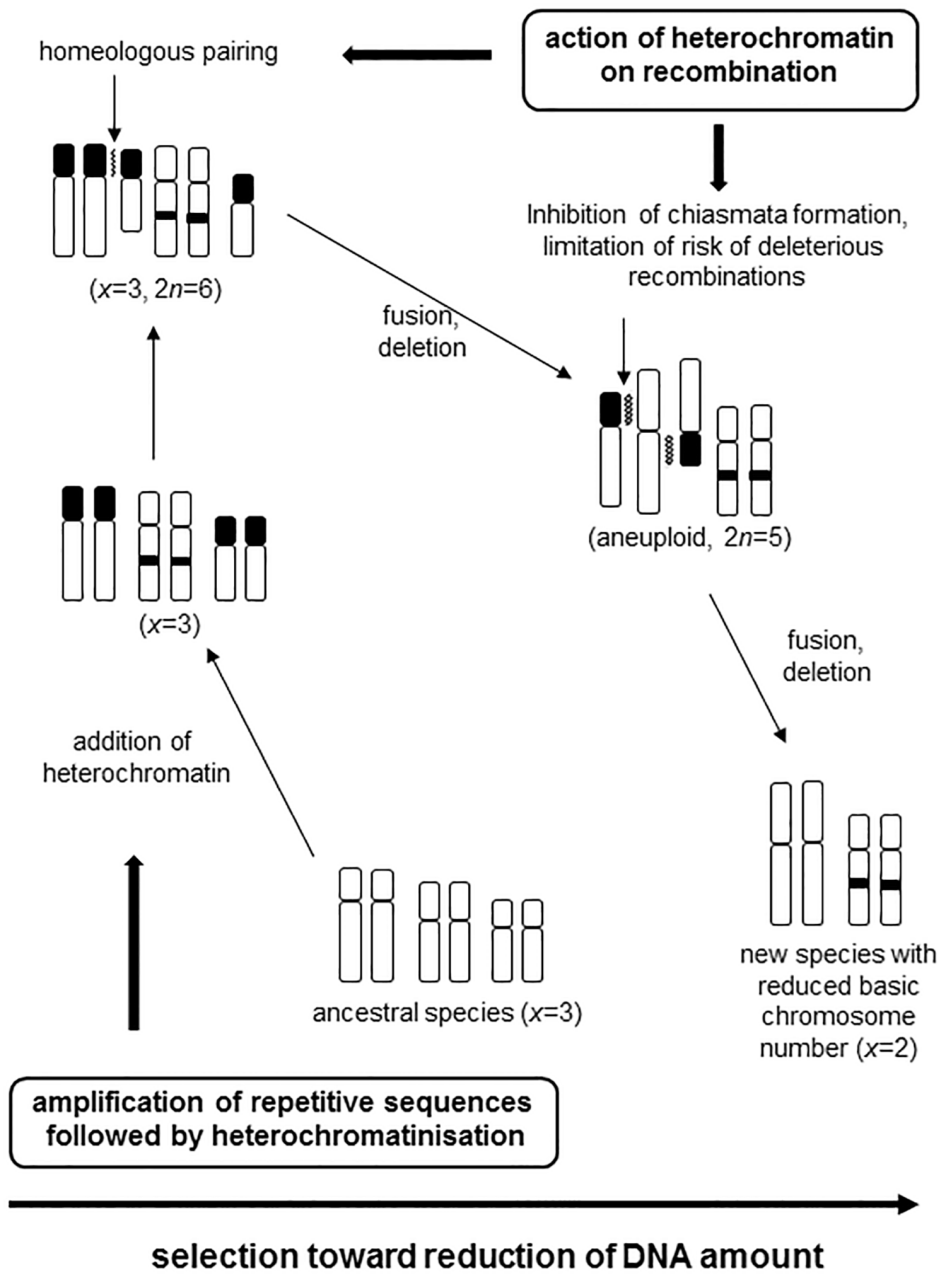
Hypothetical schema of the implication of heterochromatin in chromosomal restructuring during reduction of the basic chromosome number and decrease of DNA content.

The role of heterochromatin chromosomal restructuring during reduction of the chromosome number and decreases in DNA content was revealed for *Reichardia* in the present study. One general hypothetical schema of this evolutionary process is proposed (Fig 5).

Evolution by decreasing dysploidy requires a transitory homeologous state (Fig 5). The probability of a chromosome rearrangement is relatively low. Thus, it is unlikely that both chromosomes in a pair are subject to the same change at the same time. This homeologous state is generally considered as a deleterious state (heterozygous disadvantage). The genetic models which describe this kind of transition frequently involve the role of population structuring [79]. The karyotype polymorphism and the high frequency of homeologous karyotypes in *R*. *macrophylla*, an endemic species with a fragmented distribution area [10, 12], may be considered as arguments supporting this hypothesis.

Another evolutionary pattern in the karyotype of the genus *Reichardia* are the position (terminal or intercalary, or both) and the number of secondary constrictions (SC)—the diploid set of chromosomes in *R*. *dichotoma* presents six SC, but only four have been observed in *R*. *macrophylla*, *R*. *tingitana* and *R*. *gaditana*, with two in *R*. *picroides* and *R*. *intermedia* (Fig 2).

#### Neutral (unspecific), GC and AT-rich heterochromatin distribution

In our previous studies [10, 23], Giemsa C-banding revealed different distribution patterns of constitutive heterochromatin for five *Reichardia* species (Table 6). An increase in heterochromatin was observed in *R*. *tingitana* and *R*. *gaditana* and a decrease was observed in *R*. *picroides*. In the latter, heterochromatin is present only in centromeric regions and satellites, while other species also possess intercalary and terminal heterochromatic bands. DAPI counterstaining after FISH, in which there is a denaturation of the DNA as during C-banding technique, demonstrates the constitutive heterochromatin. Conventional DAPI staining without denaturation reveals regions of DNA rich in AT bases. Thus, the DAPI staining after FISH essentially confirmed C-banding data, revealing a different distribution of constitutive heterochromatin for each *Reichardia* species. Most of the C-bands were DAPI positive except those associated with NORs and some intercalary bands which were GC-rich and CMA^+^ (Table 5; Fig 1G, 1G’ and 1H). C-bands in SC (NORs) were always CMA positive and DAPI negative. Presence of unspecific or GC-rich heterochromatin and SCs fragility facilitates chromosome breakages at these sites and favors restructuring or rearrangement of the chromosomes. During this process the loss of heterochromatin blocks (entire chromosome arms in *R*. *gaditana* and *R*. *tingitana*) and SCs contribute to the reduction of chromosome number and genome size (Fig 5).

#### Changes in number, position and organisation of 35 and 5S rRNA genes

The particular organisation of overlapping 5S and 35S rRNA genes in *R*. *dichotoma* and *R*. *tingitana* has already been observed in numerous plants [80, 81], and for certain genera this colocalisation is the predominant pattern of rRNA genes, as is the case of *Artemisia*. In this Asteraceae genus, the colocalisation was first observed at cytological level [82, 83, 84] and then validated by molecular techniques [85]. Our cytological observations for two *Reichardia* species (*R*. *dichotoma* and *R*. *tingitana*) should be also verified by the DNA fibre mapping technique and by molecular methods, which we plan to use in future investigation of these taxa.

In *R*. *dichotoma*, 35S and 5S rRNA genes are colocalised in intercalary SC (Fig 1A’), while those in *R*. *macrophylla* are separated on different chromosome pairs (Fig 1B’). This disposition could be explained by a break in the intercalary SC of *R*. *dichotoma* followed by a translocation and inversion on two other chromosomes in the terminal position in *R*. *macrophylla* (Fig 2). In *R*. *tingitana*, 35S and 5S colocalised (Fig 1C’) while in *R*. *gaditana* these two rDNA families were located on the same chromosome arm of pair V (another 35S locus is located in chromosome pair VIII) (Fig 1F’) and separated on different chromosomes pairs in *R*. *picroides* (Fig 1G”). Changes in organisation and position of rRNA genes in *Reichardia* species were also followed by the reduction of the number of 35S and 5S loci from 3 to 1 per diploid genome. All these changes indicate substantial restructuring during dysploidy, suggesting that the process occurred over a long period of time.

#### Genome downsizing and reduction of total chromosome length and pollen size with decreasing dysploidy

Genome size and pollen size (E and P) were closely correlated in this dysploid series (Table 3). The most important genome downsizing was observed between the species with 2*n* = 18 and 2*n* = 16. Pollen size decreases perceptibly with the reduction of the basic chromosome number. Whereas the relationship between polyploidy and pollen size has been abundantly reported [86, 87, 88], it is, to our knowledge, the first time that the correlation between pollen size and dysploidy has been established. However, the positive correlation detected between 2*n* and pollen size loses its significance when considering the phylogenetic signal, suggesting that it could rather reflect a shared evolutionary history. Further analyses on an extended sampling of dysploid lineages are necessary to could shed light on the relation between dysploidy and pollen size.

#### Genome downsizing and the cell cycle: Evolutionary forces at genomic level

Fundamental properties of the cell cycle are modified by variations in the DNA amount [89, 90, 91]. For example, rapid cell division is needed to facilitate a short life cycle, for which a small nuclear DNA amount is favoured [92, 93]. By this means, natural selection is acting on the DNA amount (at the genomic level); this process can be identified as a main evolutionary force determining the pattern of heterochromatin content. This trend is verified in *Reichardia*, where *R*. *dichotoma*, *R*. *albanica* and *R*. *macrophylla*, the three species with the highest DNA amount are perennial, while the others species show a tendency toward reduced genome size and shorter life cycle.

The increase in the heterochromatic content of the two intermediate species (*R*. *tingitana* and *R*. *gaditana*) in an overall context of reduction in genome size, rDNA loci number and shorter life cycle could appear paradoxical. However, the expansion of heterochromatin area, while resulting from purely molecular processes involving the amplification of certain types of tandemly repeated sequences [94], also multiplies chromosomic regions particularly sensitive to chromosome breakages and as such favours genome restructuring that can led to genome size decrease.

## Concluding remarks

*Reichardia* constitutes a model genus for studies on genome evolution, since it presents three basic chromosome numbers for only ca. 10 taxa. This study has shown that descending dysploidy was coupled with a high genomic dynamism involving decrease in genome size, changes in heterochromatin pattern, and modifications of the location and organisation of ribosomal genes. By facilitating translocations, and especially the centric fusions frequently observed during descending dysploidy, chromosome breakage in heterochromatin area was highlighted as an important contributor to genome restructuring. In *Reichardia*, dysploidy is accompanied with pollen size reduction, a trend that should be further addressed in an extended taxonomic sampling.
